# Percutaneous Drainage for Aortic Graft Infection Post-aneurysm Repair: A Viable Option?

**DOI:** 10.1177/15385744221075136

**Published:** 2022-02-18

**Authors:** Sean A. Kennedy, M. Katharine Kennedy, Thomas F. Lindsay, John Byrne, Arash Jaberi, Wayne L. Gold, KongTeng Tan, Sebastian Mafeld

**Affiliations:** 1 Division of Vascular & Interventional Radiology, Joint Department of Medical Imaging, University Health Network and Mount Sinai Hospital, 7938 University of Toronto, Toronto, ON, Canada; 2 Division of Vascular Surgery, Department of Surgery, 7989 University of Toronto, Toronto, ON, Canada; 3 Division of Infectious Diseases, Department of Medicine, 33540 University of Toronto, Toronto, ON, Canada

**Keywords:** aortic aneurysm repair, aortic graft infection, percutaneous drainage

## Abstract

**Purpose:**

Non-operative management of aortic graft infection is usually only considered in a palliative context. We describe the safety, efficacy, and clinical outcomes of percutaneous drainage of aortic graft infections (AGI) following either open or endovascular repair of aneurysmal disease.

**Methods:**

Twelve consecutive patients (11 males, 1 female, mean age 72.7 ± 10.3 years, age range 52-88 years) between January 2010-July 2020 who underwent percutaneous drain insertion in either an infected aortic sac or periaortic abscess cavity following endovascular or open surgical graft repair were identified. Patient and procedural characteristics as well as clinical outcomes were determined.

**Results:**

Of the 12 patients who underwent percutaneous drain insertion, five (41.7%) had undergone open abdominal aneurysm repair, one (8.3%) open thoracoabdominal aneurysmal repair, and six (50%) endovascular abdominal aneurysm repairs. Drain size ranged from 10-20 French. All were inserted under ultrasound (US), computed tomography (CT), and/or fluoroscopic guidance. Median duration of drain placement was 55.2 days (range 3-174). Five patients (41.7%) had the drain in place as a stabilizing bridge until or after definitive surgical explantation and aortic reconstruction. Seven patients (58.3%) were managed with drain placement and antibiotic therapy without surgical intervention. Six (50%) were alive at the most recent time of follow-up (median, 732 days, range 166-1650 days). Three patients (25%) died during follow-up with causes including erosion of aortic reconstruction into sigmoid colon, unrelated chronic obstructive pulmonary disease exacerbation, and severe *clostridium difficile* colitis and pseudomonal pneumonia (median 1244 days, range 992-1597 days). Three (25%) patients were lost to follow-up. No drain-related complications were noted.

**Conclusion:**

Percutaneous drainage of AGI following endovascular or open aneurysm repair is a safe and viable management option either as a temporizing measure as a bridge to surgical graft explantation or as a non-surgical therapy for long term management.

## Introduction

Aortic graft infections (AGIs) following endovascular aneurysm repair or open surgical repair represent complex clinical scenarios. Definitive management of AGI conventionally requires combined antibiotic therapy, surgical explantation of graft material, aggressive debridement of the surgical bed, and reconstruction of the involved aortic segment with or without extra-anatomical bypass.^1,2^ Unfortunately, there is high morbidity and mortality associated with this approach.^3-5^ Complex medical comorbidities often preclude definitive surgical explantation. While some guidelines do recognize percutaneous drainage as a treatment strategy, data guiding its use and outcomes are extremely limited.^1,2^ Several small case series have described successful percutaneous drain placement in the context of AGI.^4,6-9^ At our institution, in select patients, we have utilized percutaneous drainage of AGI as a bridge to surgical explantation or alternatively as definitive treatment combined with antibiotic therapy. We aim to identify safety, efficacy, and clinical outcomes of percutaneous drainage of aortic/periaortic sac infections following either open or endovascular repair of thoracic and/or abdominal aortic aneurysmal disease.

## Materials and Methods

### Subject Selection and Study Design

University Health Network Research Ethics Board approval (Approved Study ID # 20-5461.0) was obtained to perform this study. We performed a retrospective chart review of all consecutive patients managed with percutaneous drainage of AGI from January 2010-July 2020 at our large quaternary vascular surgery center. Inclusion criteria were as follows:

(A) Infected aortic sac or periaortic abscess cavity
(B) Prior endovascular or open surgical graft repair of thoracic and/or abdominal aortic aneurysmal disease
(C) Underwent primary percutaneous drain insertion via either fluoroscopic and/or ultrasound US and/or computed tomography CT guidance

No patients underwent percutaneous drainage if aorto-enteric fistula was suspected. All patients were deemed to have an AGI based on the Management of Aortic Graft Infection Collaboration (MAGIC) definition for definite AGI (Table 1) with at least one major and one minor criteria from the categories of clinical, radiologic, and/or laboratory findings.^10^ A multidisciplinary care team involving vascular surgery, infectious diseases, and interventional radiology was involved in planning the management approach for all patients.

**Table 1.** MAGIC Criteria for Definition of AGI.^10^

	Clinical	Radiology	Laboratory
Major criteria	-Pus (confirmed by microscopy) around graft in aneurysm sac at surgery -Open wound with exposed graft or communicating sinus -Fistula development -Graft insertion in an infected site	Peri-graft fluid on CT scan ≥ 3 months after insertion -Peri-graft gas on CT scan ≥ 7 weeks after insertion -Increase in peri-graft gas volume on serial imaging	-Organisms recovered from an explanted graft -Organisms recovered from intra-operative specimen -Organisms recovered from percutaneous aspirate of peri-graft fluid
Minor criteria	-Localized clinical features of AGI -Fever ≥38°C with AGI as most likely cause	-Other suspicious imaging findings such as aneurysms expansion, pseudoaneurysm; activity on FDG PET; nuclear medicine studies	-Blood culture(s) positive and no apparent source other than AGI -Abnormally elevated inflammatory markers with AGI as most likely cause

### Procedural Details and Follow-up

All drain insertions were performed by 8 interventional radiologists at our institution. Using standard aseptic technique, a suitable trajectory was determined based on prior cross-sectional imaging which included anterior, lateral, and posterior approaches. Standard Seldinger technique was used to place an 18G trocar needle and wire into the aortic sac itself or peri-aortic collection using ultrasound and/or CT and/or fluoroscopic guidance. Following this, a dilator followed by standard Cope loop drains were placed in all patients with the size selection at the interventionalist’s discretion. Fluid was aspirated and sent for culture and sensitivity. Contrast injection under fluoroscopy and/or CT and/or US imaging was used to confirm appropriate position within the collection cavity. Drainage catheters were left to bag drainage. If the drainage catheters became blocked or demonstrated poor drainage, they were checked under fluoroscopy and upsized or changed as needed. All patients were followed as either inpatients or outpatients by vascular surgery. Drains were typically removed at time of surgical explantation or in non-surgical patients when patients were doing clinically well and drain output was minimal. In non-surgical patients, most continued on lifelong suppression antibiotic therapy. Co-management occurred with infectious diseases.

### Data Collection and Statistical Analysis

Primary outcomes of interest pertained to safety and efficacy, including procedural-related complications, duration of drain placement, in-hospital mortality, and mortality during follow-up. Baseline patient characteristics as well as aneurysm repair and microbiologic data were tabulated in Microsoft Excel (Redmond, USA). Descriptive statistics including mean, median and standard deviation are reported for this case series.

## Results

Twelve consecutive patients (11 males, 1 female, mean age 72.7 ± 10.3 years (range 52-88)) met inclusion criteria. All patients had CT findings which demonstrated a fluid collection (+/− gas locules) either surrounding aortoiliac graft material or within the residual aneurysm sac itself. Patient/procedural characteristics as well as clinical outcomes are delineated in Table 2. All patients met the MAGIC definition for definite AGI (Table 1) with at least one major and one minor criteria.

**Table 2.** Patient, Procedural, Microbiology, and Outcome Summary.

Patient #	Gender	Age	Site of Initial Aneurysm Repair	Open/EVAR	Surgical Explant or Medical Management	Drain Placement	Drain Placement Imaging Guidance (CT, Fluoro, US)	Maximal Drain Size (French)	Complications Related to Drain Placement	Drain Culture (Blood Culture if Drain Culture Negative)	Duration of Drain Placement (days)	In-Hopsital Mortality	Mortality During Followup?	Followup Duration Available (Days)	Antibiotic Status
1	Male	72	Abdominal	Open	Surgical explant	Right retroperitoneum/aortic sac (initial drain fell out and reinserted 13 days later)	US/Fluoro	14	None	*Enterobacter Cloacae* and bacteroides fragilis	121	No	Alive	1650	Ciprofloxacin metronidazole 4.5 months after aortic reconstruction
2	Male	74	Abdominal	EVAR	Medical	Left periaortic	CT/Fluoro	12	None	No growth (no growth)	42	No	Alive	732	Moxifloxacin 12 months after percutaneous drain insertion
3	Male	68	Abdominal	Open	Surgical explant and repair of ADF	Left periaortic/abdominal	CT/Fluoro	12	None	Enterobacter aerogenes and enterococcus faecium	54	No	Unrelated death (pneumonia/COPD exacerbation)	1244	No long term suppressive antibiotics
4	Male	64	Abdominal	Open	Surgical explant	Left periaortic (drain placed after explant for residual collection)	CT/Fluoro	12	None	Candida albicans	9	No	Related death (erosion of femoral vein graft into sigmoid colon)	1597	No long term suppressive antibiotics
5	Female	64	Thoracoabdominal	Open	Medical	Left retrotracheal/periaortic mediastinal	US/CT/Fluoro	12	None	*Pseudomonas Aeruginosa* and coagulase negative staphylococcus	3	No	LTFU	LTFU	Indefinite ciprofloxacin/metronidazole
6	Male	76	Abdominal	Open	Medical	Left psoas/peri-iliac	CT/Fluoro	10	None	*Klebsiella Pneumoniae* and enterobacter cloacae	48	No	LTFU	LTFU	Indefinite clavulin
7	Male	88	Abdominal	EVAR	Medical	2 drains right groin abscess followed by left aortic sac	CT/Fluoro	10	None	*Staphylococcus Aureus*	59	No	Alive	744	Indefinite cephalex, fluconazole
8	Male	81	Abdominal	EVAR	Surgical explant	Left periaortic	CT/Fluoro	10	None	*Escherichia Coli*, parabacteroides distasonis, bacteroides thetaiotaomicron	8	No	LTFU	LTFU	6 weeks IV meropenum and fluconazole after which patient LTFU
9	Male	74	Abdominal	EVAR	Medical	Right periaortic	CT/Fluoro	12	None	Propionibacterium acnes	90	No	Alive	1371	Indefinite penicillin
10	Male	52	Abdominal	EVAR	Medical	Left periaortic (outside study period had had previous drainage 2.5 years prior)	US	12	None	No growth (no growth)	46	No	Related death (severe C. Difficile colitis)	992	Indefinite amoxicillin-clavulanic acid
11	Male	88	Abdominal	EVAR	Medical	Left aortic sac	CT/Fluoro	12	None	No growth (no growth)	174	No	Alive	174	Indefinite amoxicillin-clavulanic acid, ciprofloxacin
12	Male	71	Abdominal	Open	Surgical explant	Left periaortic/abdominal	US	20	None	*Bacteroides Fragilis*	8	No	Alive	166	Indefinite amoxicillin-clavulanic acid

Regarding the original surgical procedures, five patients had undergone open abdominal aneurysm repair (41.6%), one open thoracoabdominal aneurysmal repair (8.3%), and six endovascular abdominal aneurysm repairs (50%). Three patients (25%) had the drain in place as a stabilizing bridge until surgical explantation and neo-aortic reconstruction. One patient had the drain in-situ to manage a collection post-surgical explantation (8.3%) and 8 patients (66.6%) were managed solely with drain placement and antibiotic medical therapy; of which only one (8.3%) has had indefinite long-term drain placement. Median duration of drain placement was 55.2 days (range 3-174).

No drain-related complications or in-hospital mortality were encountered. Of the nine patients (75%) with complete follow-up data, total median follow-up was 992 days. Three patients (33%) died at a median of 1244 days post-drain placement (range 992−1597 days). Two deaths (22.2% of the patients for which there was follow-up information) were deemed related to underlying AGI (one from erosion of a femoral vein graft into sigmoid colon and one related to severe *Clostridioides difficile* colitis secondary to lifelong suppressive antibiotic therapy) and one was deemed unrelated (pneumonia and COPD exacerbation). With respect to the remaining six patients, they were alive at a median 732 days of available follow-up (range, 166−1650 days).

**Figure 1.**
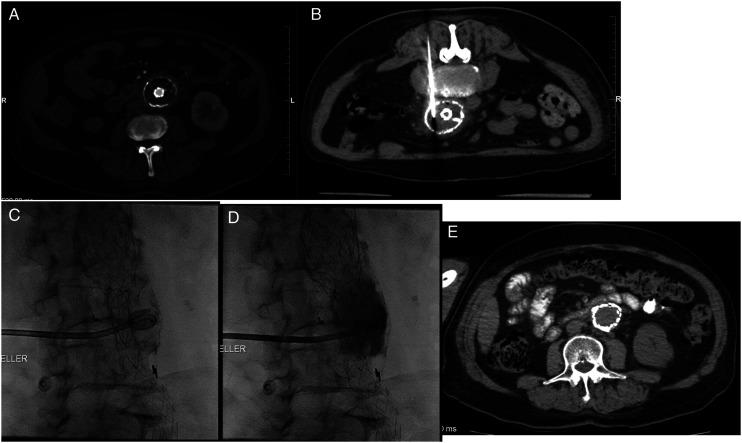
A: Prior infrarenal aorto uni-iliac stent graft. Gas-containing, fluid filled aneurysm sac which had demonstrated progression in size compared to previous (not depicted). B-D: CT- and fluoroscopy-guided placement of 10Fr drain into aortic sac via left posterior approach. E: Drain was removed 59 days following placement. CT at approximately 2 years followup demonstrates no evidence of residual collection or peri-aortic inflammatory change.

Representative imaging findings of percutaneous drainage over time can be found in Figure 1A-E.

## Discussion

Aortic graft infection is uncommon with an approximate incidence of 0.2-0.3%.^11-14^ Randomized controlled trial and patient registry data have not demonstrated a significant difference in infection risk between open versus endovascular repair.^11,12^ Nonetheless, AGI either post-endovascular or open surgical aneurysm repair are extremely challenging clinical scenarios that often result in poor outcomes. Oftentimes, patients are too frail to undergo the gold-standard morbid operative management of surgical graft explantation, aortic reconstruction with aggressive peri-graft debridement and therefore require less invasive means of management as a temporizing bridge either to surgical repair or as long−term management. Our data reveals that percutaneous drainage is a safe, effective, and viable option for the management of these complex patients. Available literature on AGI is predominantly weighted towards reporting outcomes following aggressive surgical management. Kahlberg et al^5^ present a 2016 systematic review on management of thoracic AGI. This review included 233 patients with a mix of both surgical and endovascular thoracic aortic grafts. While they reported a trend towards lower 1-year mortality with graft explantation compared to graft preserving therapies (OR .3 95% CI .1−1, *P* = .56), this did not reach significance. Li et al^3^ present a 2018 systematic review on the management of both thoracic and abdominal endovascular stent-graft AGIs including 402 patients. Of these patients, 10% received conservative treatment compared to 90% receiving surgical explantation and reconstruction in addition to antibiotic therapy. The surgical group had a higher survival rate compared to the conservative management group (58% vs 33%, *P* = .002). Most patients who underwent conservative treatment in the included studies, however, did not receive percutaneous drainage, but rather received antibiotic therapy alone with other medical supportive measures management and/or other ancillary procedures such as esophageal stenting. Many of these patients also had suspected aorto-enteric fistulas. Accurately identifying an aorto-enteric fistula on CT imaging can be challenging. A distinct advantage of percutaneous drainage with contrast injection is that it can accurately detect aorto-enteric fistulas. No active aorto-enteric fistulas were suspected at the time of drain insertion in our study.

Outside of case reports, available literature on percutaneous management of AGI is limited to a few small case series with a total of less than 50 patients (Table 3).^6-10^ Our experience favorably compares to the majority of available literature with no reported in-hospital mortality or 30-day mortality and no drain-related complications. Additionally, 67% of patients were alive at a median follow-up of 2 years post-drainage placement; with only 2 deaths attributable to AGI sequelae (22.2%). While graft explanation is the only definitive therapy, this series has demonstrated that percutaneous drainage and long-term suppression antimicrobial therapy is a valid treatment option, particularly for patients where operative risk is prohibitive.

**Table 3.** Summary of Case Series which Reported Outcomes for Percutaneous Drainage for the Management of AGI.

Study Author and Year	Open/EVAR	30-Day Mortality	Other
Batt et al 2012 (6)	Open	2/5	Further details not reported
Bélair et al 1998 (7)	Open	0/11	9/11 septic process resolved
			4/9 percutaneous drainage only
			5/9 drainage followed by surgical explantation
Martins et al 2019 (8)	EVAR	0/3	3/3 drainage only
Pryluck et al 2010 (9)	EVAR	0/3	1/3 complete resolution with percutaneous drainage only
			1/3 extra-anatomic bypass and graft explantation
			1/3 debridement and fistula repair with preserved endograft
Lyons et al 2013 (4)	EVAR	0/3	1/3 percutaneous drainage only died 15 months post
			1/3 TEVAR + percutaneous drainage died 10 months post
			1/3 TEVAR + percutaneous drainage died 3 months post

Kaneda et al and Igari et al presented five and six patients, respectively, with AGI all of which were managed without explantation.^15,16^ All patients underwent debridement, followed by drain placement with irrigation and drainage. Kaneda et al^15^ utilized irrigation solutions of saline containing .4% povidone-iodine solution intermittently and/or saline containing antibiotics continuously. Igari et al utilized 500 mL .02% gentian violet saline solution once daily for irrigation. Excellent results were obtained with only 1 death secondary to sepsis.^16^ Irrigation was not performed in our study and its role represents another potential avenue for future investigation. Antibiotic irrigation, however, may not provide additional benefit if patients are maintained on chronic suppressive antibiotic therapy.

Current guidelines regarding the management of AGI do briefly mention percutaneous drainage. Specifically, the 2018 Society of Vascular Surgery guidelines state:

*“Percutaneous drainage and antibiotic therapy have been suggested for patients unfit to undergo open repair.”* ^*2*^

More recently, the 2020 European Society for Vascular Surgery guidelines also indicate percutaneous drainage as a potential treatment option but cite a 30-day mortality rate of 40%, describing its role as *“controversial.”*^1^ Our 30-day mortality was 0%. It is our hope that our experience may counter some of this *“controversy”* regarding the use of percutaneous drainage as a valid management option, either as a definitive therapy or as a bridge to surgery. In our series the majority (5/6) of the EVAR patients we treated medically with no explanation while 4/6 of the open aneurysm repairs were treated with treated by explanation following percutaneous drainage. Radical surgical treatment was performed on those whose infection persisted and were open surgical candidates. Drainage prior to explanation has the advantage of pre-operative control of sepsis allowing more elective intervention without the hazards associated with acute systemic infection.

The present study does have some limitations. Most notably, this is a single center retrospective study. While this is one of the larger studies available in the literature, our experience is still limited to that of a small case series. Furthermore, our study was conducted at a quaternary care high-volume vascular surgery center with strong multidisciplinary care teams in place to optimize patient selection for drainage. Such results and experiences may not be generalizable to smaller centers, although percutaneous drainage is a readily available treatment in most hospital settings. Aortic graft infection is quite variable in its presentation and heterogeneous with respect to microbial etiologies as well as the extent of graft/perigraft involvement. We grouped together various microorganisms and anatomical sites of involvement ranging from the aortic sac itself to the peri-aortic graft and surrounding tissues. All infections are unlikely to behave in the same manner and further larger studies are required to delineate optimal patient selection and timing for percutaneous drainage versus surgical management. Furthermore, the diagnosis of AGI itself can be controversial; however recent literature has sought to clarify this with defined radiological, clinical and laboratory criteria.^10^ Use of positron-emission tomography (PET) is increasingly being advocated for in guidelines to evaluate for the presence of AGI and to differentiate from non-infectious inflammatory pathologies.^1,10^ Positron-emission tomography is not routinely funded for AGI evaluation in our healthcare system. While some may view this as a study limitation, with a multidisciplinary evaluation of clinical, microbiological and imaging findings, we are confident all included patients had true AGI. *In the three cases in this series where cultures were negative, the first had a positive white cell scan but negative cultures, the second had continued aneurysm expansion without endoleak and drainage of puruluent material and the third had a systemtic sepsis with positive blood cultures one month prior to aortic drain insertion which yielded purulent material.* It is our experience that patients who are culture negative had suppressive antiobiotics prior to aortic drainage.

Recent efforts have been made to better understand this challenging pathology with the creation of the MAGIC.^10^ This is a national database based in the United Kingdom, dedicated to evaluating AGI. We commend the leaders of this effort as the understanding of AGI requires true multidisciplinary management; however, balancing the high mortality of endograft explantation with antibiotic therapy and percutaneous drainage remains challenging and necessitates highly individualized decision making.

In summary, percutaneous drainage is a safe and effective means to manage patients with AGI when coupled with antimicrobial therapy who are either unfit for surgery or require temporizing measures prior to definitive surgical explantation.
